# Microstructure, chemical composition, and dielectric response of CaCu_3_Ti_4_O_12_ ceramics doped with F, Al, and Mg ions

**DOI:** 10.1016/j.heliyon.2023.e18523

**Published:** 2023-07-21

**Authors:** O.Z. Yanchevskii, O.I. V'yunov, T.O. Plutenko, A.G. Belous, V.V. Trachevskii, I. Matolínová, K. Veltruská, V. Kalinovych, Ye Lobko

**Affiliations:** a Dept. Solid State Chemistry, V. I. Vernadsky Institute of General and Inorganic Chemistry of the National Academy of Science of Ukraine, Acad. Palladin Ave. 32/34, 03142, Kyiv, Ukraine; b G.V. Kurdyumov Institute of Metal Physics of the National Academy of Science of Ukraine, Acad. Vernadskii Ave. 36, 03680, Kyiv, Ukraine; c Dept. Surface and Plasma Science, Faculty of Mathematics and Physics, Charles University, V Holešovičkách 2, 180 00, Prague, Czech Republic

**Keywords:** CaCu_3_Ti_4_O_12_ (CCTO), Dielectric permittivity, Energy-dispersive X-ray spectroscopy (EDX), X-ray photoemission spectroscopy (XPS), Electron paramagnetic resonance (EPR)

## Abstract

Ceramics with nominal chemical composition CaCu_3_Ti_4_O_12_ (CCTO), CaCu_3_Ti_3.96_Al_0.04_O_11.96_F_0.04_ (CCTOAF), and Ca_0.98_Mg_0.08_Cu_2.94_Ti_3.96_Al_0.04_O_11.96_F_0.04_ (CCTOMAF) were prepared by the solid-state reactions technique. Using SEM, EDX, XPS, EPR, NMR, and complex impedance spectroscopy, the microstructure, elements distribution, chemical composition of grains and grain boundaries, and the dielectric response of ceramics were investigated. In the ССТО, CCTOAF, and CCTOMAF series, the average grain size increases, the degree of copper segregation at the grain boundaries is inversely related to grain size, and the dielectric loss decreases from 0.071 to 0.047 and 0.030, respectively, while dielectric permittivity ε′ at 1 kHz is 5.6 × 10^4^, 7.1 × 10^4^, and 4.3 × 10^4^, respectively. Additives of Al, Mg, F and milled particles (ZrO_2_, Al_2_O_3_, and SiO_2_) can either partially introduce into the perovskite structure or form low-melting eutectics at the grain boundaries, causing abnormal grain growth. The presence of copper ions in various oxidation states, as well as evidence of exchange spin interactions between them, was confirmed in all samples.

## 1 Introduction

For two decades, electrically heterogeneous ceramics CaCu_3_Ti_4_O_12_ (CCTO) has attracted the attention of researchers due to its anomalously high dielectric response (ε′ = 10^4^-10^5^) in a wide temperature range (100–600 K) without any structural transitions [1]. This has opened up opportunities for using CCTO in the fabrication of efficient energy storage devices, the evolution of solid-state capacitors, flexible and stretchable ultra-sensitive wide-angle small capacitive pressure sensors for electronic wearable equipment (electronic skin), smart electronics, and visible light-induced photoelectrochemical and photocatalytic activity devices [2,3]. However, the loss tangent (tan *δ*) of CCTO ceramics is generally higher than the standard level for practical applications as ceramic capacitors; that is why investigations are being made to reduce the value of tan *δ*. For CCTO-based ceramics, the maximum value of ε′ is ∼400 000 [1], whereas the theoretical value of ε′ for CCTO according to the Clausius-Mossotti relation should be as low as ∼49 [4]. The internal barrier layer capacitor (IBLC) at the boundary between semiconductor grains with double Schottky barriers is the generally accepted model [5,6]. A double Schottky barrier consists of a negative charge layer at the grain boundary surrounded by a symmetrical depletion layer [7]. The barrier width is inversely proportional to temperature exponentially, and a smaller width can significantly improve the permittivity of CCTO samples. However, the IBLC model does not explain why high dielectric responses are not observed in isostructural analogs and why ССTO single crystals demonstrate so high dielectric responses. Progress has been made in explaining the latter through the discovery of internal domains within CCTO grains [8] and the formation of a nanoscale barrier layers capacitance (NBLC) model. This model suggests that planar defects in the bulk of grains act as potential barriers to charge transfer between semiconductor domains [9]. The dielectric response is also affected by the existence of space charge regions near the electrodes, which is accounted for by the surface barrier layers capacitance (SBLC) model [10].

The structure of CCTO (A′A″_3_B_4_O_12_) with space group Im $\bar{3}$ can be derived from the simple cubic perovskite structure ABO_3_ by separating Ca and Cu ions in A-sites with different coordination numbers (CN). Namely, Ca ions are located in the A′ site with CN 12, and Cu ions are located in the plane-square A″-site with CN 4, which tilts the TiO_6_ octahedra. Ti^4+^ ions are displaced from the center of the oxygen octahedra along the (001) direction and form one-dimensional, antiparallel, mutually independent dipole chains of finite length [11]. Due to the displacement of Ti^4+^ ions by ∼0.04 Å, CCTO exhibits a spontaneous polarization of 0.4 μC/cm^2^ [12]. The nanoscale disordering of the Ca and Cu positions due to the fluctuation of the TiO_6_ octahedra gives the contribution to the giant dielectric response of CCTO. Ca/Cu disordering was detected by quantitative electron diffraction (QED), extended X-ray absorption fine structure (EXAFS), and X-ray photoelectron spectroscopy (XPS) methods [[13], [14], [15], [16]].

In CCTO ceramics, two types of polaron conductivity are observed, namely n-type Ti^4+^ + е^–^ ↔ Ti^3+^, and p-type Сu^2+^ + h^+^ ↔ Cu^3+^ [17]. Even visible light stimulates transitions of Cu^2+^ and Ti^4+^ ions in CCTO to excited states, Cu^3+^ and Ti^3+^ with photogeneration of e^–^/h^+^ pairs. The low-energy transition takes place between the hybridized Cu 3 d - O 2p valence band and the unoccupied Cu 3 d band, whereas the high-energy transition occurs between the valence band and the Ti 3 d conduction band [3]. According to the defect model [17], the domain boundaries are Cu vacancies, and the internal grain boundaries are Ti vacancies. In such a case, the ratio of grain size to domain size is proportional to the ratio of contents of Cu vacancies to Ti vacancies and should be variable during sintering. This is one of the reasons for the existence of the optimal duration and temperature of ceramic sintering. Annealing CCTO ceramics in an oxygen-enriched atmosphere slightly decreases dielectric response, ε′ and decreases the dielectric loss, tan *δ* [18], while quenching ceramics increases ε′ and decreases tan *δ* [19].

Previously, we have shown that simultaneous doping with fluorine and aluminum is promising for improving the dielectric properties of CCTO ceramics [20]. In addition, magnesium doping may also be helpful but literature data on the structural features of such compounds are contradictory. Namely, the introduction of magnesium only into copper sites in CaCu_3-*x*_Mg_*x*_Ti_4_O_12_, depending on the experimental conditions, either leads to an increase in volume V of the unit cell [21] or does not affect it [22]. It is unexpected that substitution in both sites (Cu and Ca) of the systems CaCu_3-*x*_Mg_*x*_Ti_4_O_12_ and Ca_1-*x*_Mg_*x*_Cu_3_Ti_4_O_12_ results in a decrease in V at *x* = 0.01 and the increase in V at *x* ≥ 0.03 compared to the undoped sample (x = 0) [23]. The authors of the work [23] claimed that Mg^2+^ ions replace ions in A or B sites of A′A″BO_3_ structure depending on the concentrations of Mg doping, *x*.

In this work, we apply SEM, XRD, EDX, XPS, EPR, NMR, and complex impedance spectroscopy to determine the dependence of dielectric characteristics on the real chemical composition of phases inside and on the surface of CCTO grains (undoped and doped with Al, F, and Mg).

### 1.1 Experimental method

Ceramics samples of nominal composition CaCu_3_Ti_4_O_12_ (CCTO), CaCu_3_Ti_3.96_Al_0.04_O_11.96_F_0.04_ (CCTOAF), and Ca_0.98_Mg_0.08_Cu_2.94_Ti_3.96_Al_0.04_O_11.96_F_0.04_ (CCTOMAF) were synthesized by the solid-state reactions technique. Analytical grade СuC_2_O_4_⋅nH_2_O, CaCO_3_, Mg_2_OCO_3_, CaF_2_, extra pure TiO_2,_ Al(NO_3_)_3_ were used as starting reagents. First, the CaCO_3_:TiO_2_ mixture with a molar ratio of 1:1 was ball-milled with corundum (Al_2_O_3_) balls and isopropanol for 6 h. Then the mixture was dried and heat-treated at 1100 °C for 6 h to form CaTiO_3_ [24]. Stoichiometric quantities of CaTiO_3_ and other reagents corresponding to the nominal compositions of CCTO, CCTOAF, and CCTOMAF were grounded with isopropanol and ZrO_2_ bullets using a planetary mill Retch PM100 for 4 h at 300 rpm. The resulting suspensions were dried at 100 °C and heat-treated at 920 and 960 °C for 12 h with intermediate grinding for 0.5 h in an agate mortar. A 5% aqueous solution of polyvinyl alcohol was added to the synthesized powders at a ratio of 1:20. The mixture was then sieved through a 150-mesh nylon sieve and ground again. Pellets with a diameter of ∼9 mm and a thickness of ∼3 mm were pressed at 100 MPa. The pellets were sintered at 1100 °C for 10 h with a heating and cooling rate of 250 and 350 °C/h, respectively.

The phase composition of the samples was determined using a DRON-4-07 X-ray diffractometer with a Cu tube operating at a 40 kV and 20 mA voltage and current respectively [25]. Scanning electron microscopy (SEM) was performed on a MIRA 3 microscope (Tescan GmbH, Czech Republic) operating at 30 kV electron beam energy in secondary electron mode (SE). The elemental compositions of the samples were studied through energy-dispersive X-ray spectroscopy (EDX) using a Bruker XFlash detector coupled with SEM. 200 μm^2^ area or point EDX analysis was performed to monitor changes in the composition of volume and secondary phases, respectively. The values of concentrations of individual elements in a given sample were calculated with an error of about 10%. The core-level spectra of chemical elements on the ceramic surfaces were examined by X-ray photoelectron spectroscopy (XPS) by a SPECS Phoibos 150 hemispherical electron-energy analyzer (SPECS Surface Nano Analysis GmbH, Germany) with a multichannel detector and an Al *K*α X-ray source in ultrahigh vacuum (UHV) conditions. The XPS spectra were fitted with a Gaussian-Lorentzian function and Shirley background using KolXPD software [26]. Archimedes’ method was used to determine the density of the samples. To measure electrical characteristics, ceramic samples were polished and electrodes were applied by firing silver paste at 600 °C. Complex impedance was measured by a 1260 Impedance/Gain-Phase Analyzer (Solartron Analytical) at 1 V ac voltage in the frequency range from 20 Hz to 1 MHz. For complex impedance data processing, ZView software (Scribner Associates Inc., USA) was used. The values of ε′ and tan *δ* were calculated from complex impedance data and the dimensions, namely the thickness of the sample and the area of the electrode. The measurement error in the frequency range 10^3^–10^5^ Hz did not exceed 500 for ε′ and 0.002 for tan *δ* [27].

The electron paramagnetic resonance (EPR) spectra were obtained on an Elexsys E580 EPR spectrometer (Bruker Biospin, Rheinstetten, Germany) with an operating frequency in the X-band range (9400 MHz) at 295 K. The g-factor of signals was calculated according to the relationship: h × ν_0_ = g × β × H, where h is Planck constant, ν_0_ is the microwave frequency, β is the Bohr magnetron and H is the magnetic field strength. The nuclear magnetic resonance (NMR) spectra were recorded on a Bruker Avance 400 spectrometer. ^63^Cu NMR spectra were obtained at a sweep width of 2 MHz at 106.34 MHz using 16384 data points. Each spectrum was obtained in the accumulation mode using a single-pulse sequence with 1 MHz bandwidth and 0.1 s delay. The pulse repetition time and pulse width were 0.5 s and 4 μs, respectively. Typically, 2 000 000 transients were collected for all samples. The chemical shift (*δ*) of ^63^Cu NMR spectra are referenced to external standards of Cu metal and KCuO_2_ [28]. The decomposition of each spectrum was performed with a pseudo-Voigt function to obtain the best fit.

## 2 Results and discussion

**Fig. 1:**
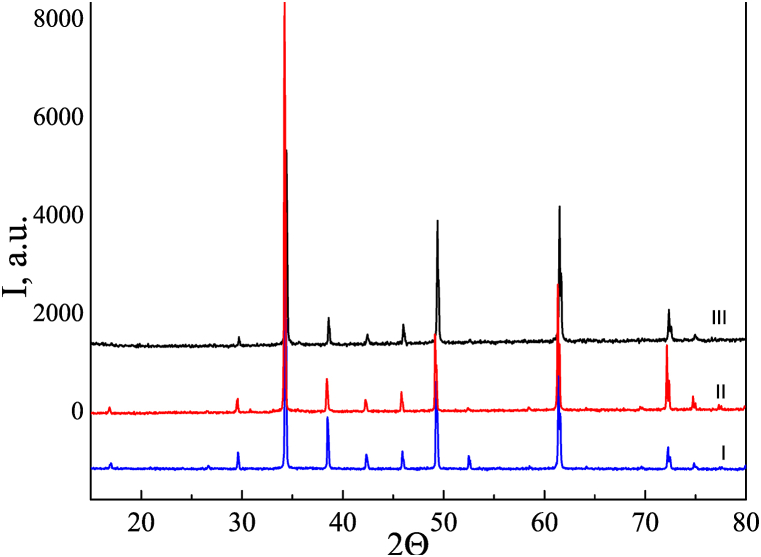
X-ray diffraction patterns of ceramics ССТО (І), CCTOAF (ІІ), and CCTOMAF (ІІІ), sintered at 1100°С/10 h.

Fig. 1 shows that all samples are single-phase and belong to the space group Im $\bar{3}$ (204); their unit cell parameters are: *a* = 7.3930 (7) Å, V = 404.1 (1) Å^3^ (CCTO); *a* = 7.3939 (6) Å, V = 404.2 (1) Å^3^ (CCTOAF); *a* = 7.3902 (5) Å, V = 403.62 (8) Å^3^ (CCTOMAF). According to the literature, the simultaneous substitution of O^2−^ (1.40 Å) by F^−^ (1.33 Å) and Ti^4+^ (0.605 Å) by Al^3+^ (0.535 Å) [29] should lead to a decrease in the unit cell volume. But the unit cell volume of CCTOAF is slightly larger than that of CCTO. This can be explained by a decrease in cation charge to compensate for the decrease in anion charge which occurs when oxygen is replaced by fluorine. This should result in an increased contribution from ions with large radii, such as Ti^3+^ (0.67 Å) or Cu^1+^ (0.60 Å). In Ca_0.98_Mg_0.08_Cu_2.94_Ti_3.96_Al_0.04_O_11.96_F_0.04_, Mg^2+^ ions partially (∼¾ of the total amount) enter copper sites with a coordination number 4 and have the same ionic radius (0.57 Å). Other Mg^2+^ ions (∼¼) enter calcium sites with a coordination number 8 and have a smaller ionic radius (0.89 Å) than Ca^2+^ ions (1.34 Å). As a result, the unit cell volume should decrease, which is confirmed by the experiment and agrees with the literature data [30].

**Fig. 2:**
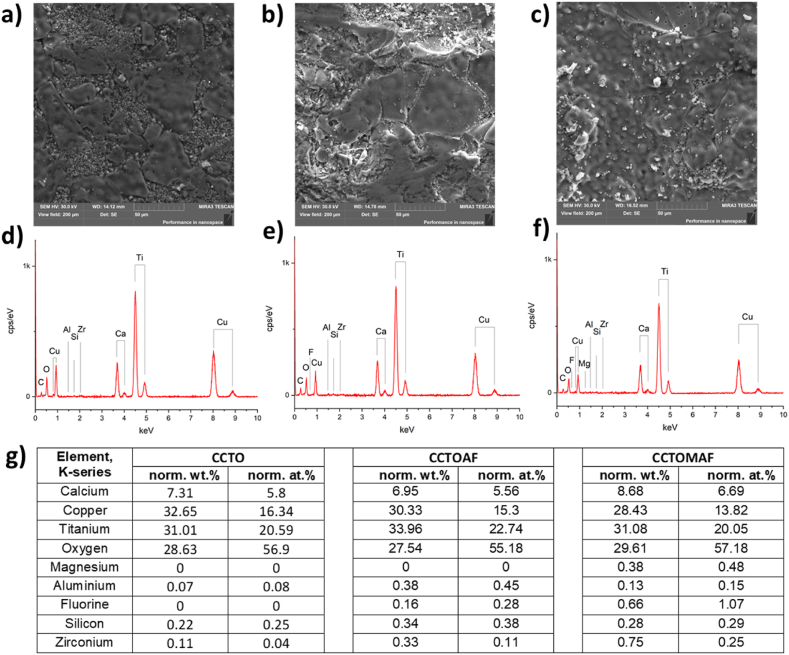
SEM images (view field 200 μm) and corresponding EDX spectra of ceramics ССТО (а,d), CCTOAF (b,e), and CCTOMAF (c,f). Quantitative analysis (g) of the EDX spectra presented (d–f).

The relative densities of CCTO, CCTOAF, and CCTOMAF ceramics are 94.0%, 93.0%, and 93.5% respectively. SEM shows (Fig. 2) that all ceramics are characterized by a bimodal structure and contain large (20–100 μm) and small (0.5–4 μm) grains. The smallest (0.5–2 μm) and the largest grains (80–150 μm) are observed in CCTO and CCTOMAF, respectively (Fig. 2a and c). For CCTO ceramics synthesized by the solid-state reactions technique, the bimodal distribution of grain size is often observed [31,32]. EDX spectra of ceramics CCTO, CCTOAF, and CCTOMAF (Fig. 2 d-g) show the presence of Si, Zr, and Al. Thus grinding medium ZrO_2_, Al_2_O_3_, and SiO_2_ along with copper oxide can form low-melting eutectic and cause abnormal grain growth. The introduction of Al_2_O_3_, СaF_2_, and MgO (CCTOAF and CCTOMAF) can increase the amount of liquid phase at the grain boundaries, reduces its melting temperature, and increases the number of large grains and their size [[33], [34], [35], [36], [37], [38], [39]]. Therefore, the addition of fluorine, aluminum, and especially magnesium promotes grain growth.

**Fig. 3:**
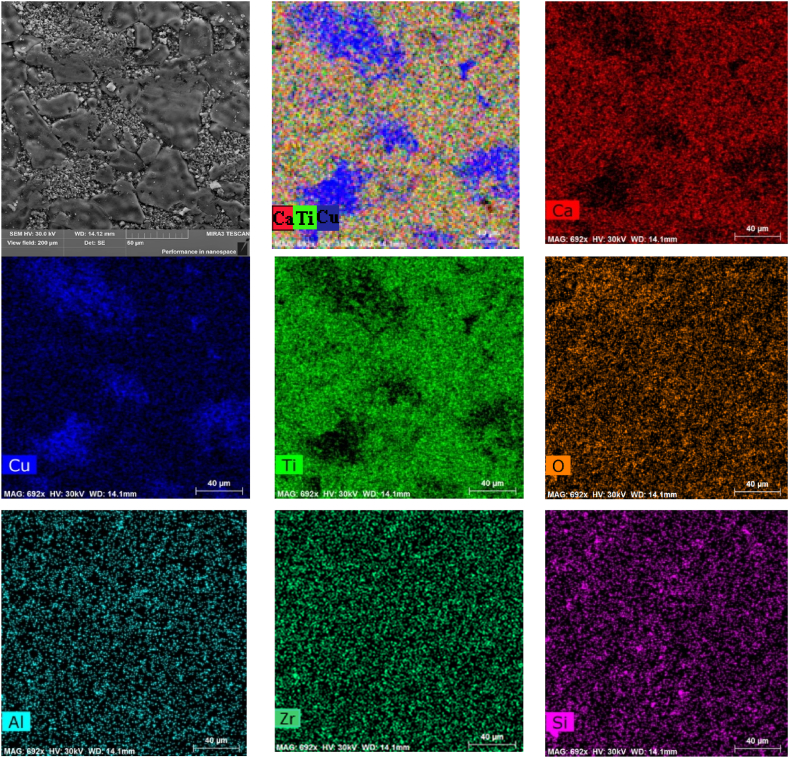
SE image (top left) of CCTO ceramics with corresponding EDX color elements map (top center), and maps of the individual elements. Scale 40 μm. (For interpretation of the references to color in this figure legend, the reader is referred to the Web version of this article.)

**Fig. 4:**
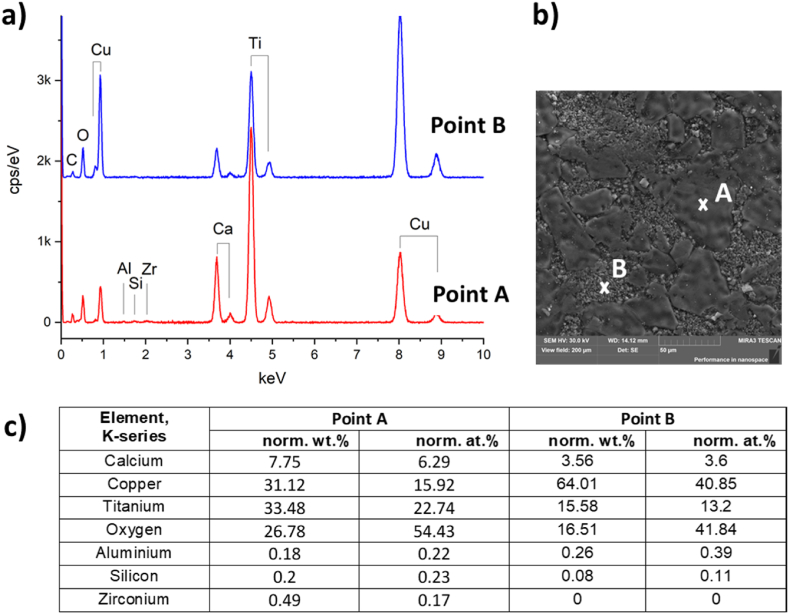
EDX spectra (a) and the content of the elements (c) at points A and B of CCTO ceramics (b). Elemental composition calculated from the spectra in (a): CaCu_2.48_Ti_3.95_Zr_0.01_Al_0.01_Si_0.02_O_11.5_ (point A); CаCu_11.5_Ti_3,99_Si_0.05_Al_0.02_Zr_0.01_O_20.4_ (point B).

Fig. 3 shows images of surface morphology (taken in SE mode) and EDX maps of elements distribution in CCTO ceramics. In CCTO, segregation of Cu and deficiency of Ca and Ti only on the smallest grains d_g_ ≤ 5 μm is observed. In addition, the presence of Zr, Al, and Si is observed due to the use of ZrO_2_ and corundum bullets and an agate mortar. Fig. 4 shows the EDS spectra of two various points (A and B) of CCTO ceramics, and the weight and atomic contents of elements in these areas. In both areas, traces of silicon, aluminum, and zirconium are observed. Fig. 4,c shows that the composition of the coarse grain of the main phase (point A) can be described as CaCu_2.48_Ti_3.95_Zr_0.01_Al_0.01_Si_0.02_O_11.5_ and that of the fine grain (point B) as CаCu_11.5_Ti_3.99_Si_0.05_Al_0.02_O_20.2_. This indicates that composition with Cu deficit is formed on the surface of coarse CCTO grains, all Zr, as well as part of Al and Si, is included in the perovskite structure, and the surface of fine grains of the composition CаCu_11.5_Ti_3.99_Si_0.05_Al_0.02_O_20.2_ can be described as follows: CаCu_3_Ti_4_O_12_ -8.5СuO - 0.05SiO_2_ -0.01Al_2_O_3_. Thus, in the local areas between particles CuO, Al_2_O_3_ and SiO_2_, liquid-phase eutectics can form and promote abnormal grain growth [40,41].

**Fig. 5:**
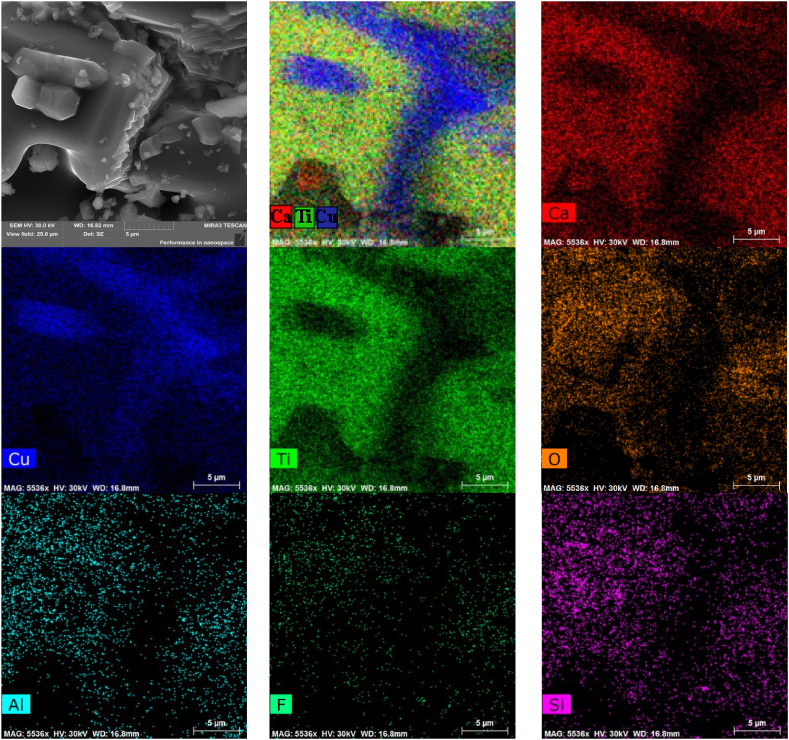
SE image (top left) of CCTOAF ceramics with corresponding EDX color elements map (top center), and maps of the individual elements. Scale 5 μm. (For interpretation of the references to color in this figure legend, the reader is referred to the Web version of this article.)

Fig. 5 shows the surface morphology (in SE mode) and EDX maps of element distribution in CCTOAF. As seen in Fig. 5, Cu also segregates on fine grains (≤3–6 μm) and areas of Cu segregation are accompanied by a deficiency of Ca and Ti. The EDX analysis reveals the expected presence of F, in addition to Al and Si. These elements are relatively evenly distributed throughout the volume of ceramics, regardless of the grain size.

**Fig. 6:**
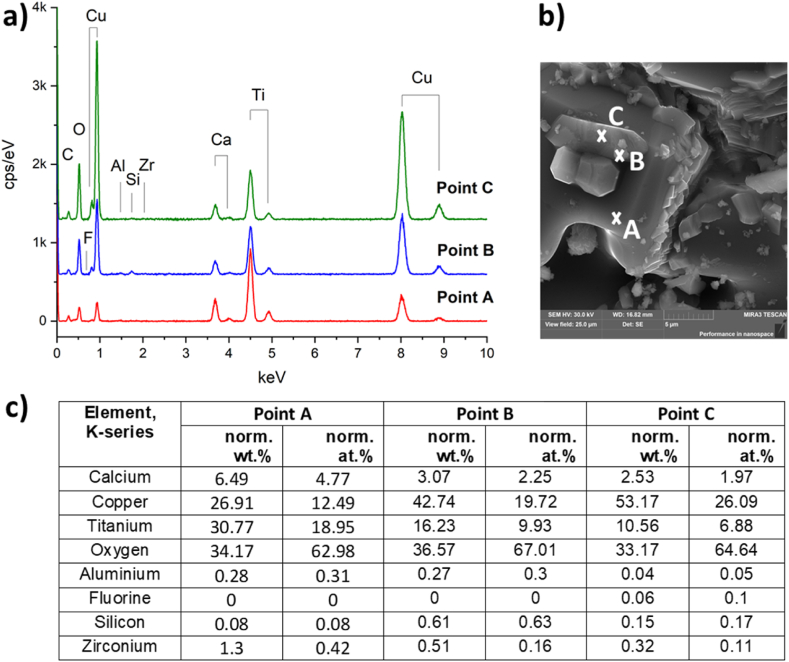
EDX spectra (a) and the content of the elements (c) of ССТОAF ceramics (b). Elemental composition calculated from the spectra in (a): CaCu_2.36_Ti_3.67_Zr_0.02_Al_0.01_Si_0.03_O_11_ (point A); CaCu_8.88_Ti_4_Zr_0.1_Al_0.08_Si_0.18_O_19_ (point B); CaCu_15_Ti_3.95_Zr_0.05_Al_0.01_Si_0.05_F_0.05_O_24_ (point C).

Fig. 6 shows the EDS spectra of three various points of CCTOAF ceramic: A is located in coarse grain, B and C in fine grains. The coarse CCTOAF grain is Cu- and Ti-deficient with a small amount of Al and Si and unexpectedly contains no fluorine according to the chemical formula CaCu_2.36_Ti_3.67_Zr_0.02_Al_0.01_Si_0.03_O_11_. The fine grains (point B) are enriched in Cu, Si as follows CaCu_8.88_Ti_4_Zr_0.1_Al_0.08_Si_0.18_O_19_. Another fine-grained part (point C) is more enriched in copper and contains fluorine according to the chemical formula CaCu_15_Ti_3.95_Zr_0.05_Al_0.01_Si_0.05_F_0.05_O_24_. Therefore, it can be assumed that the F^−^ ions do not replace O^2−^ ions in the A′A″_3_B_4_O_12_ structure, but accumulate at the boundaries of fine grains and participate in liquid-phase eutectics. In coarse-grained areas, where a single-phase solid solution is formed, the experimentally determined total B-site substitution limit for Si^4+^ and Al^3+^ ions does not exceed 0.04.

**Fig. 7:**
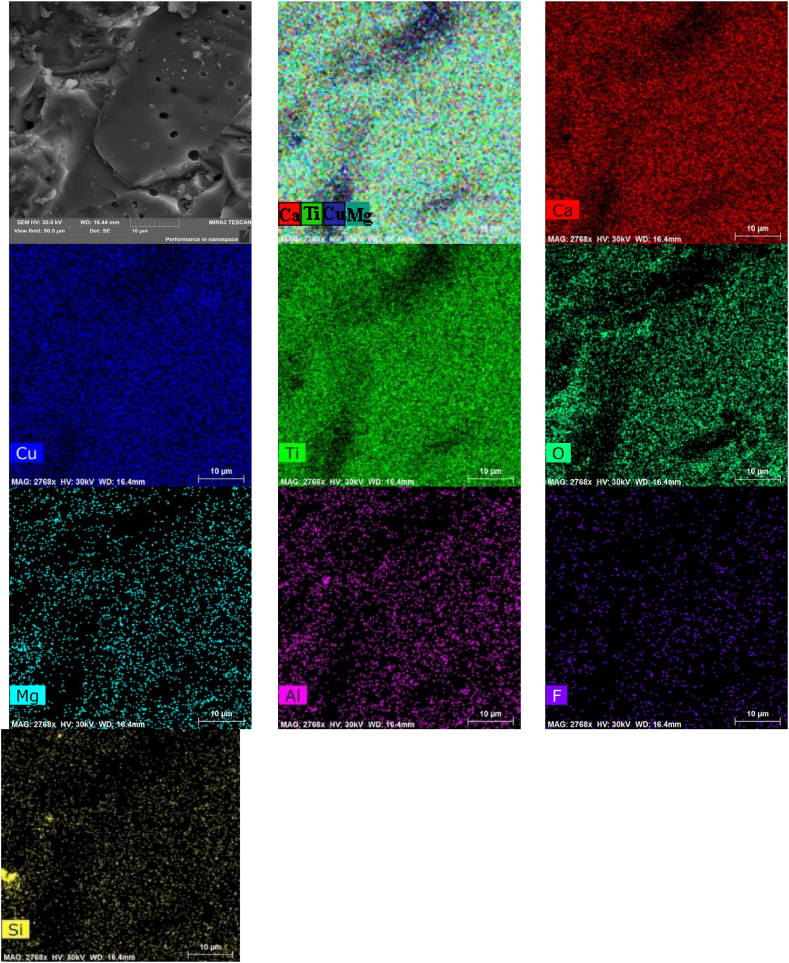
SE image (top left) of CCTOMAF ceramics with corresponding EDX color elements map (top center), and maps of the individual elements. Scale 10 μm. (For interpretation of the references to color in this figure legend, the reader is referred to the Web version of this article.)

Fig. 7 shows the surface morphology (in SE mode) and EDX maps of element distribution in Mg-doped ceramic CCTOMAF. Areas of Cu segregation disappeared, and fine grains coalesced. Therefore, the segregation of copper (CuO) is inversely proportional to the grain size. This can be explained on the one hand by the larger specific surface of fine grains and a shorter diffusion distance of copper ions to the grain boundary, and on the other hand by the inclusion of segregated copper into the volume of coarse grains. The process of abnormal grain growth involves the growth of larger grains at the expense of their smaller neighbors, leading to the successive elimination of finer grains and an increase in average grain size [42]. Fig. 7 shows that Mg, Ti, and Ca are uniformly distributed, therefore Mg-containing solid solutions are formed. The local areas with high content of Si and Al at the grain junctions are observed. Traces of Zr are also observed on the maps of element distributions for CCTOAF and CCTOMAF (not shown).

**Fig. 8:**
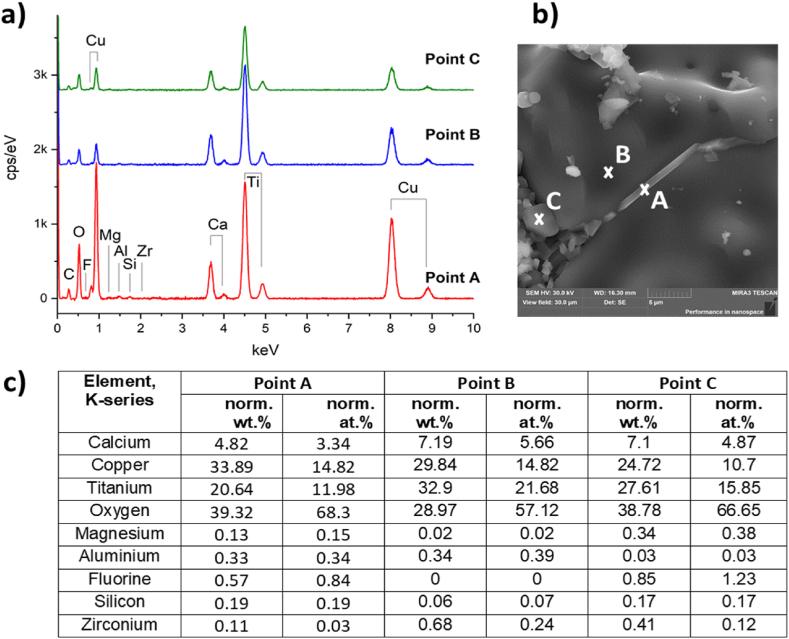
EDX spectra (a) and the content of the elements (c) at points A, B, and C of ССТОМAF ceramics (b). Elemental composition calculated from the spectra in (a): Ca_0.98_Mg_0.06_Cu_4.9_ Ti_4.05_Al_0.07_Si_0.08_F_0.06_O_14.35_ (point A); Ca_0.98_Mg_0.02_Cu_2.74_Ti_4.1_Al_0.03_O_14.3_ (point B); Ca_0.98_Mg_0.08_Cu_2.6_Ti_4.0_Al_0.07_Si_0.06_F_0.07_O_11.9_ (point C).

Fig. 8 shows EDX spectra and chemical composition of the intergranular phase (point A), coarse (point B), and fine grains (point C) in CCTOMAF ceramics. The intergranular phase has a slight excess of copper -Ca_0.98_Mg_0.06_Cu_4.9_Ti_4.05_Al_0.07_Si_0.08_F_0.06_O_14.35_. The composition of coarse grains (point B) is close to nominal, with a slight copper and magnesium deficiency, and without fluorine -Ca_0.98_Mg_0.02_Cu_2.74_Ti_4.0_Zr_0.045_Al_0.03_Si_0.006_O_14.3_, which indicates the formation of single-phase solid solution. The composition of fine grains (point C) is characterized by a high content of magnesium, aluminum, silicon, and fluorine Ca_0.98_Mg_0.08_Cu_2.6_Ti_4.0_Al_0.07_Si_0.06_F_0.07_O_11.9_, which indicates the participation of these elements in the formation of low-melting eutectics. Noteworthy is the increase in the limit of substitution of Al^3+^ ions for titanium positions in the perovskite structure from ∼0.015 for CCTOAF to ∼0.03 for CCTOMAF. Silicon and aluminum do not compete for entry into the titanium site for CCTOMAF, since Si^4+^ ions form stable magnesium silicates (Mg_2_SiO_4_) at temperatures 1000–1100 °C [43]. That is why the introduction of magnesium not only promotes grain growth but also decreases the charge of the Ti sublattice in the presence of Al additives.

Combined neutron and synchrotron diffraction full-profile analysis shows that during the sintering of CCTO ceramics, Cu^2+^ ions leave their sites and diffuse to the grain boundaries, Ti^4+^ ions pass into vacant Cu^2+^ positions, and in the grain bulk, a semiconductor copper-deficient solid solution Ca^2+^(Cu^2+^_1-*x*_Ti^4+^_*x*_)_3_(Ti^4+^_4-6*x*_Ti^3+^_6*x*_)O_12_ is formed [44]. However, the replacement of Ti^4+^→Cu^2+^ should lead to the appearance of vacancies in the titanium position. Therefore, no more than half of the copper vacancies can be replaced by titanium ions and only calcium positions remain nondeficient. In the formation of semiconducting grains and copper-enriched grain boundaries, several stages can be distinguished. The first stage is the diffusion of both Cu^2+^ and O^2−^ ions to the grain boundary:

(1) CaCu_3_Ti_4_O_12_=Ca^2+^(Cu^2+^_3-*x*_)(Ti^4+^_4_)O_12-*x*_ + *x*CuО

The copper deficiency inside the grain results in an overstoichiometry of titanium and calcium. However, Eq. (1) shows that simply filling copper positions with Ti^4+^ ions without additional loss of oxygen cannot lead to the formation of Cu^1+^ and Ti^3+^ ions. The latter are formed according to the scheme Cu^1+^ + Ti^4+^ → Cu^2+^ + Ti^3+^. At the next stage, in addition to the diffusion of Cu^2+^ and O^2−^ ions to the grain boundaries, oxygen is lost. This leads to the formation of Cu^1+^ or Ti^3+^ in the bulk:

(2) Ca^2+^(Cu^2+^_3-*x*_)(Ti^4+^_4_)O_12-*x*_ = Ca^2+^(Cu^2+^_3-3*x*_Cu^1+^_*x*_Ti^4+^_*x*_)(Ti^4+^_4-*x*_)O_12-2.5*x*_+1/2*x*Cu_2_О +1/2*x*О_2_↑

(3) Ca^2+^(Cu^2+^_3-*x*_)(Ti^4+^_4_)O_12-*x*_ = Ca^2+^(Cu^2+^_3-2*x*_Ti^3+^_*x*_)(Ti^4+^_4-*x*_)O_12-2.5*x*_+1/2*x*Cu_2_О +1/2*x*О_2_↑

If part of the copper at the grain boundaries evaporates, and part of the oxygen losses is compensated during the cooling stage, the ions Cu^3+^ in grain bulk are formed:

(4) Ca^2+^(Cu^2+^_3-*x*_)(Ti^4+^_4_)O_12-*x*_ = Ca^2+^(Cu^2+^_3-3*x*_Cu^3+^_*x*_Ti^4+^_*x*_)(Ti^4+^_4-*x*_)O_12-1.5*x*_ + 1/2*x*Cu_2_O

According to Eq. (2) and (3), 1.5 mol of O^2−^ should be lost for the formation of 1 mol of Сu ^+^ or Ti^3+^, and according to Eq. (4), 0.5 mol should be lost for the formation of 1 mol Cu^3+^. It is known that at 1100 °C, the amount of reversible and irreversible losses of oxygen and copper from the CCTO powder is 1 wt% and 1.5 wt% respectively [45]. Reversible losses imply the reoxidation of grain boundaries and near-boundary regions during the cooling of sintered ceramics. Taking into account that losses from pressed billets are always much less than from powders, the content of Cu^1+^, Ti^3+^, and Cu^3+^ ions in CCTO ceramics can be written as follows:

(5) [Cu^1+^] + [Ti^3+^] + [Сu^3+^] « 1.5 × [M_Cu_ /1.5M_O_] × 2.5 wt%

where M_Cu_ and M_O_ are the molecular weight of copper and oxygen respectively. Eq. (5) shows that the content of ions responsible for conductivity in ССTO is much less than 15 wt%. The calculated content of elements in different valence states should be treated critically, as the analysis method can introduce a systemic error. Specifically, the higher contents of Cu^3+^, Cu^+^, and Ti^3+^ ions on the ceramic surface, as determined by XPS under visible light and high vacuum [3,46], can be attributed to the oxidation/reduction of ions under measurement conditions. However, this data is useful for comparative assessment of the chemical state of elements on the surface of samples.

**Fig. 9:**
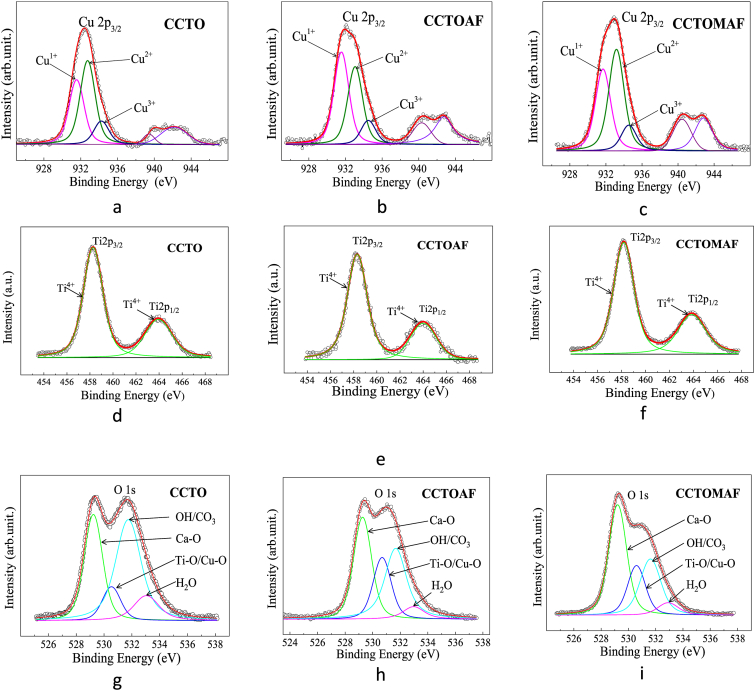
XPS core level spectra of Cu 2p_3/2_ (a–c), Ti 2p (d–f), O 1s (g–i) of ceramic CCTO, CCTOAF, and CCTOMAF.

Fig. 9 shows the core-level XPS spectra of Cu 2p_3/2_, Ti 2p, and O 1s regions of surfaces of ССТО, CCTOAF, CCTOMAF ceramics. The Cu 2*p*_*3/2*_ core-level spectra of ceramics contain peaks at 931.6, 933.4 and 934,5 eV (Fig. 9, a-c) which belong to Сu^1+^, Cu^2+^ and Cu^3+^ respectively [9,34,[47], [48], [49], [50], [51], [52], [53]]. The Cu^1+^/Сu^2+^/Cu^3+^ content ratio (in at.%) is 37.7/48.8/13.5 for CCTO, 47.3/40.3/12.4 for CCTOAF, and 39.2/48.5/12.3 for CCTOMAF, respectively. The highest content of Cu^1+^ (47.3 at.%) is observed for CCTOAF. The introduction of fluorine and aluminum into CCTO increases the content of Cu^1+^ (Fig. 9, a-f). This may indicate that, despite the results of the EDC analysis, an insignificant part of fluorine either replaces oxygen positions F^−^ → O^2−^ or contributes to the formation of oxygen vacancies and shifting the equilibrium of Eg. (2) to the right. The formation of Cu^3+^ ions and the weakly changing amount (12.3–13.5%) in the CCTO-CCTOAF-CCTOMAF series can be attributed to the irreversible loss of Cu^2+^ ions according to Eq. (4), rather than due to Al^3+^ → Ti^4+^ substitutions.

The core-level peaks of Ti 2p_3/2_ and Ti 2p_1/2_ are located in the ranges of 457–460 eV and 462–463 eV, respectively. Peaks at 458.6 and 464.1 eV (Fig. 9,d-f) belong to Ti^4+^ ions [52,54], and it is not possible to reliably determine the presence of Ti^3+^. However, normalized spectra of X-ray absorption near edge structure (XANES) for the Ti K-edge confirm the existence of a small (2–3%) amount of Ti^3+^ [34]. Therefore, Ti^3+^ ions can exist in ceramics and contribute to conductivity, since ceramic grains can exhibit semiconducting properties even with small amounts (*x* < 0.01) of oxygen loss [36].

Fig. 9, g-i shows core-level peaks of the O 1s on the surface of ceramics samples. The O 1s XPS spectra consist of four peaks: lattice oxygen at 529.2–529.3 eV(O′_latt_) and 530.5–530.7 eV (O″_latt_) related to the O^2−^ ions surrounded by Ca and Ti/Cu, respectively; chemically adsorbed oxygen caused by the surface hydroxyl (OH^−^) and carbonate (CO_3_^2−^) groups at 531.6–531.9 eV (O_chem_) and physically adsorbed H_2_O at 532.5–533.2 eV (O_phys_) [[55], [56], [57], [58], [59]]. The intensity of different forms of oxygen is different, as it is determined by both the phase composition and the specific surface area exposed to air and humidity. Among the studied samples, CTOMAF has the lowest content of chemically adsorbed oxygen in hydroxyl and carbonate groups due to the smaller surface area.

**Fig. 10:**
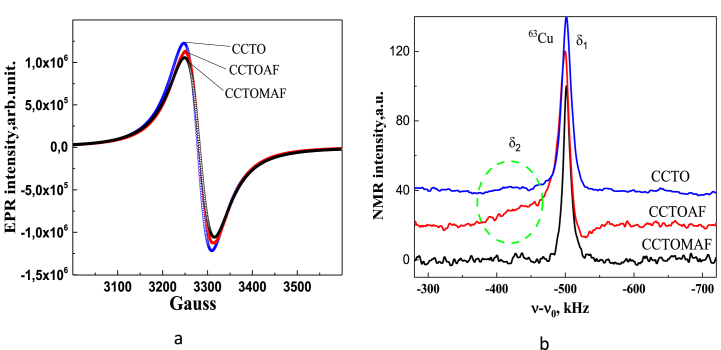
EPR (a) and NMR (b) spectra of ceramic samples CCTO (blue), CCTOAF (red), and CCTOMAF (black). (For interpretation of the references to color in this figure legend, the reader is referred to the Web version of this article.)

The EPR spectrum of CCTO, CCTOAF, and CCTOMAF samples (Fig. 10,a) shows an isotropic line of Cu^2+^ (3 d^9^, I = 3/2) ions with square planar coordination characterized by a g-factor of 2.147 and an EPR line-width of ΔH = 64 Gauss [[60], [61], [62]]. This fact indicates the significant existence of spin-exchange interactions between copper ions and suggests the formation of defects in the coordination sphere of Cu(II) ions [63]. Impurity ions in the vicinity of Cu contribute to the formation of exchange interactions Сu^2+^- O^2−^ - Cu^3+^ ↔ Сu^3+^- O^2−^ - Cu^2+^ [64,65]. This is confirmed by the detection of an additional component of signals in the ^63^Cu NMR spectrum with a chemical shift of *δ*_2_ = −4160 ppm and a half-width ν-ν_0_ = 50 kHz near the main component *δ*_1_ = −4395 ppm and a half-width ν-ν_0_ = 25 kHz (Fig. 10,b). A similar weak additional component was observed in the reference sample KCuO_2_, which contains Cu^3+^ ions. This provides additional evidence for the existence of Cu^3+^ ions in the structure. The detection of this fact requires a systematic NMR study to identify the factors affecting the formation of certain states of copper ions.

**Fig. 11:**
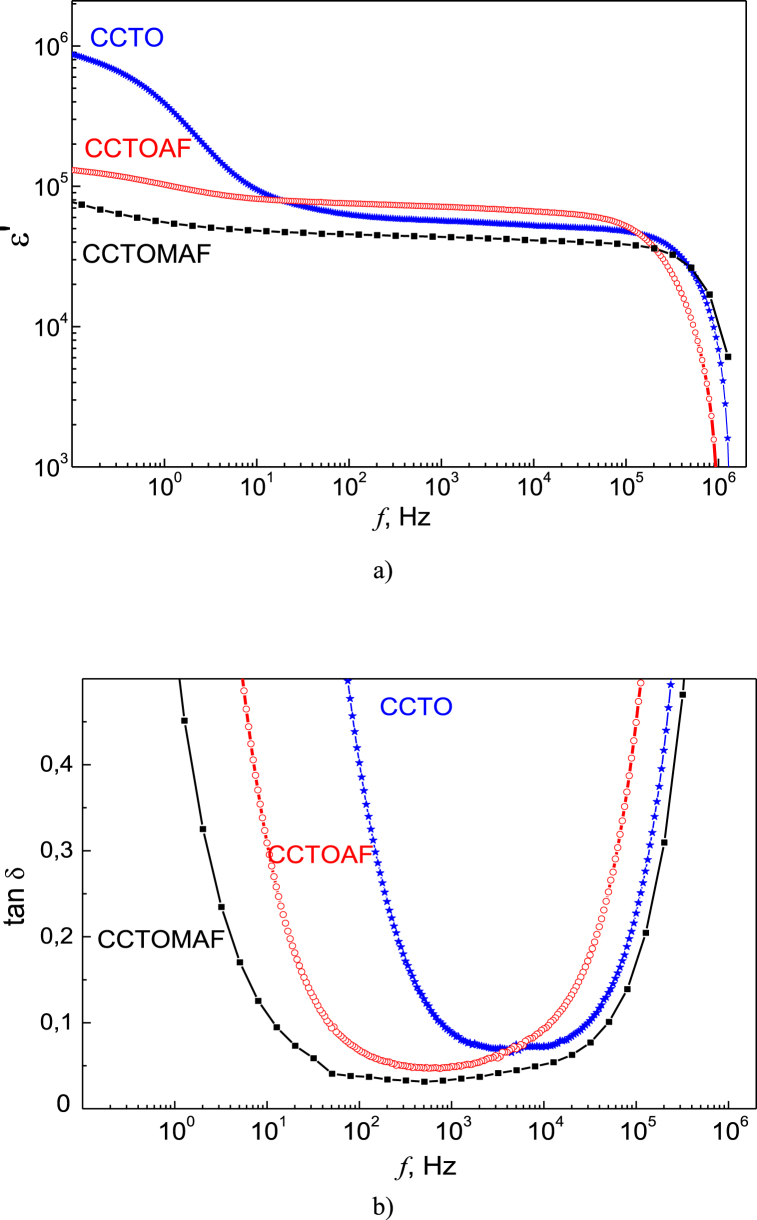
Frequency dependence of the real part of the permittivity (a) and dielectric loss (b) of ceramics ССТО (blue), CCTOAF (red), and CCTOMAF (black). (For interpretation of the references to color in this figure legend, the reader is referred to the Web version of this article.)

Fig. 11 shows the real part of the permittivity ε′ and dielectric loss tan *δ* of CCTO, CCTOAF, and CCTOMAF ceramics at room temperature as a function of frequency. Below ∼40 Hz, carrier migration causes space charge polarization without direct current conduction [66]. This effect is most noticeable in ССTO with high bimodality because the relaxation results from an uneven distribution of space charge throughout the sample [66]. The low-frequency plateau with high ε′ and sharp decline (Fig. 3a) is described by the brickwork layer form of the internal barrier layer capacitor (IBLC) model and Jonscher low-frequency dispersion [5,40]. When ceramics contains relatively large grains, in addition to the model of IBLC, the nanosized barrier layer capacitor (NBLC) model becomes essential for explaining the dielectric response. The NBLC model assumes the formation of structures based on negatively charged TiO_5_ clusters and neighboring positively charged Cu^2+^ ions (polarons). The collective action of these structures results in high permittivity up to the MHz range [40]. The permittivity at 1 kHz for CCTO, CCTOAF, and CCTOMAF samples is 5.6 × 10^4^, 7.1 × 10^4^ and 4.3 × 10^4^, respectively. At higher frequencies, relaxation of polarons of various types is observed, including those involving charged oxygen vacancies and electron hopping between Ti^3+^ and Ti^4+^ states [[66], [67], [68]], and results in the decrease of the real part of the permittivity. Fig. 11b shows that the lowest dielectric losses occur in the frequency range between the two relaxations. Additionally, dielectric loss decreases sequentially in doped CCTO: from 0.071 in CCTO to 0.047 in CCTOAF and 0.030 in CCTOMAF. The frequency range of small values of tan *δ* of doped CCTO has expanded because the grain boundary conductivity decreases, which is of practical importance [69]. Formulas are known in the literature that the values of ε′_eff_ and tan *δ* (assuming that ε_g_ ≈ ε_gb_ and ρ_g_ « ρ_gb_) relate to the characteristics of the grain boundary as follows [69,70]:

(6) ε′_eff_ ≈ ε_gb_ × d_g_ / t_gb_,

(7) tan *δ* ≈ 1/ω × ε_gb_ × ρ_gb_,

where ε_gb_ is the permittivity of grain boundaries, d_g_ is the average grain size, and t_gb_ is the grain boundary width, ρ_gb_ is the resistivity of grain boundaries. Based on Eqs. (6), (7), a change in ε_gb_ should lead to an opposite change in the values of ε′_eff_ and tan *δ*, which does not correspond to the experiment. Therefore, ε_gb_ does not significantly differ in the series CCTO - CCTOAF - CCTOMAF. Since the average grain size of CCTOMAF ceramics is larger than in CCTO and CCTOAF (cf. Fig. 2c and a, b), it can be assumed that their grain boundary width and resistivity of grain boundaries are increased. It should be noted that there is a relationship between tan *δ* and ε′, which is described by the empirical relation tan *δ* = *a* × ln (ε′) – *c*, where *a* and *c* are constants [71]. This relation shows that ceramics with both a very high dielectric constant and a very low dielectric loss cannot be prepared.

## 3 Conclusions

Undoped ceramic and ceramics doped with aluminum, fluorine, and magnesium of nominal composition CaCu_3_Ti_4_O_12_, CaCu_3_Ti_3.96_Al_0.04_O_11.96_F_0.04_, and Ca_0.98_Mg_0.08_Cu_2.94_Ti_3.96_Al_0.04_O_11.96_F_0.04_ were prepared by solid-state reactions technique. The chemical composition of the grains is different from the nominal one. The milling of grinding media and the purity of the starting reagents affect the dielectric characteristics of CCTO ceramics. It was established that the segregation of copper is inversely proportional to the grain size. Additives of fluorine, aluminum, and magnesium can either partially enter the perovskite structure or form low-melting eutectics at the grain boundaries, causing abnormal grain growth. It was supposed that the content of oxygen vacancies (and Сu^1+^, Ti^3+^) in the grain boundaries can decrease in coarse-grained ceramics due to a decrease in the grains’ surface area. As a result, the dielectric loss of CCTO ceramics has been improved. Magnesium-aluminum-fluorine modified CCTO ceramic (CCTOMAF) achieves an excellent combination of a high dielectric constant of 4.3 × 10^4^ at 1 kHz and low dielectric loss tan *δ* = 0.030. The existence of exchange spin interactions between Cu^2+^ and Cu^3+^ ions is shown. An increase in the average grain size provides a reduction in dielectric losses.

## Author contribution statement

O.Z. Yanchevskii: Performed the experiments; Analyzed and interpreted the data; Wrote the paper.

Oleg I. V'yunov: Conceived and designed the experiments; Performed the experiments; Analyzed and interpreted the data; Wrote the paper.

Anatolii Belous: Conceived and designed the experiments; Contributed reagents, materials, analysis tools or data.

Volodymyr Trachevskij: Iva Matolínová: Katerina Veltruská: Performed the experiments. Viacheslav Kalinovych: Eugenia Lobko: T.O. Plutenko: Analyzed and interpreted the data.

## Data availability statement

No data was used for the research described in the article.

## Declaration of competing interest

The authors declare that they have no known competing financial interests or personal relationships that could have appeared to influence the work reported in this paper.
